# Ras promotes cell survival by antagonizing both JNK and Hid signals in the *Drosophila *eye

**DOI:** 10.1186/1471-213X-9-53

**Published:** 2009-10-20

**Authors:** Yue Wu, Yuan Zhuang, Min Han, Tian Xu, Kejing Deng

**Affiliations:** 1 Institute of Developmental Biology and Molecular Medicine and School of Life Science, Fudan University, Shanghai 200433, PR China; 2 Department of Immunology, Duke University Medical Center, Durham, NC 27708, USA; 3 Howard Hughes Medical Institute and Department of MCDB, University of Colorado, Boulder, CO 80309-0347, USA; 4 Howard Hughes Medical Institute and Department of Genetics, Yale University School of Medicine, New Haven, Connecticut, CT 06536, USA

## Abstract

**Background:**

Programmed cell death, or apoptosis, is a fundamental physiological process during normal development or in pathological conditions. The activation of apoptosis can be elicited by numerous signalling pathways. Ras is known to mediate anti-apoptotic signals by inhibiting Hid activity in the *Drosophila *eye. Here we report the isolation of a new loss-of-function *ras *allele, *ras*^*KP*^, which causes excessive apoptosis in the *Drosophila *eye.

**Results:**

This new function is likely to be mediated through the JNK pathway since the inhibition of JNK signalling can significantly suppress *ras*^*KP*^-induced apoptosis, whereas the removal of *hid *only weakly suppresses the phenotype. Furthermore, the reduction of JNK signalling together with the expression of the baculovirus caspase inhibitor p35, which blocks Hid activity, strongly suppresses the *ras*^*KP *^cell death. In addition, we find a strong correlation between *ras*^*KP*^-induced apoptosis in the eye disc and the activation of JNK signalling.

**Conclusion:**

In the *Drosophila *eye, Ras may protect cells from apoptosis by inhibiting both JNK and Hid activities. Surprisingly, reducing Ras activity in the wing, however, does not cause apoptosis but rather affects cell and organ size. Thus, in addition to its requirement for cell viability, Ras appears to mediate different biological roles depending on the developmental context and on the level of its expression.

## Background

Programmed cell death, or apoptosis, is a fundamental physiological process in multicellular organisms. It plays a critical role in normal development where it is required for proper morphogenesis and tissue homeostasis, as well as serving a protective mechanism against extracellular pathogenic agents [1-3]. Apoptosis is also seen in pathological conditions such as when cells are deprived of survival signals. The biochemical pathway involved in apoptosis has been shown to be conserved from lower organisms, such as *Drosophila*, to mammals. The activation of apoptosis can be elicited by numerous signalling pathways.

*Drosophila *eye development is one of the best models for studying mechanisms of apoptosis [4]. The compound eye is composed of about 800 units called ommatidia. Each ommatidium has eight photoreceptor cells and six supporting cells, all differentiated from epithelial cells in the larval eye imaginal disc [5]. During late pupal development, excess cells that are not recruited for differentiation are removed by apoptosis. Thus, mutations which cause excessive or insufficient apoptosis will disrupt pattern formation during eye development and, consequently, the highly precise structure of the adult eye. Previous work has revealed two antagonizing pathways regulating apoptosis during eye development. Notch signalling is required for apoptosis [6], while the EGFR/Ras pathway is required for cell survival [7].

In *Drosophila*, Ras signalling is thought to inhibit apoptosis by antagonizing the activity of Hid, which promotes apoptosis through the degradation of the *Drosophila Inhibitor of Apoptosis Protein 1 (DIAP1) *[8]. As a consequence of Ras signalling, not only is *hid *expression reduced, but the Hid protein itself is phosphorylated and becomes inactivated [9,10]. Interestingly, although ubiquitous expression of a dominant active form of Ras could inhibit a majority of cell death that occurs normally in the fly embryo, cell death is not completely eliminated even in embryos mutant for a *hid *null allele [10,11]. This observation suggests the possibility of a Hid-independent pathway regulating apoptosis, which can be suppressed through other means besides Ras. One of the candidate signals is the PI3K/Akt pathway, which has been shown to regulate apoptosis in mammals and to be a major downstream target of activated Ras [12]. However, so far there is no evidence to support this hypothesis in *Drosophila*.

c-Jun N-terminal protein kinase (JNK) signaling is involved in the regulation of morphogenesis, cell proliferation, cell differentiation, cell migration, and apoptosis, including tumor progression and metastasis [13-15]. In the fly, JNK-induced apoptosis has an important role in the morphogenesis of the wing imaginal disc during development [1]. During eye development, the overexpression of Eiger, the *Drosophila *homolog of mammalian TNF, triggers JNK signaling causing the loss of eye tissue as a result of excessive apoptosis [16,17]. Cross-talks have been found between the Ras/MAPK and JNK pathways in regulating cell survival and apoptosis [18,19]. In cultured mammalian cells, Raf-1 has been shown to promote cell survival by antagonizing ASK1 [20], a JNK activator [21].

Here we report the isolation of a new loss-of-function *ras *allele, *ras*^*KP*^, which causes excessive apoptosis in the *Drosophila *eye. Our analysis shows that, in addition to Hid, JNK pathway plays a significant role in mediating cell death in the eye and is antagonized by Ras antiapoptotic activity. In contrast to its effect in the eye, we also show that Ras regulates organ size in the developing wing by affecting cell size, indicating the developmental output of Ras signalling is highly context-dependent.

## Results

### A new loss-of-function ras allele affects ras expression level

**Figure 1:**
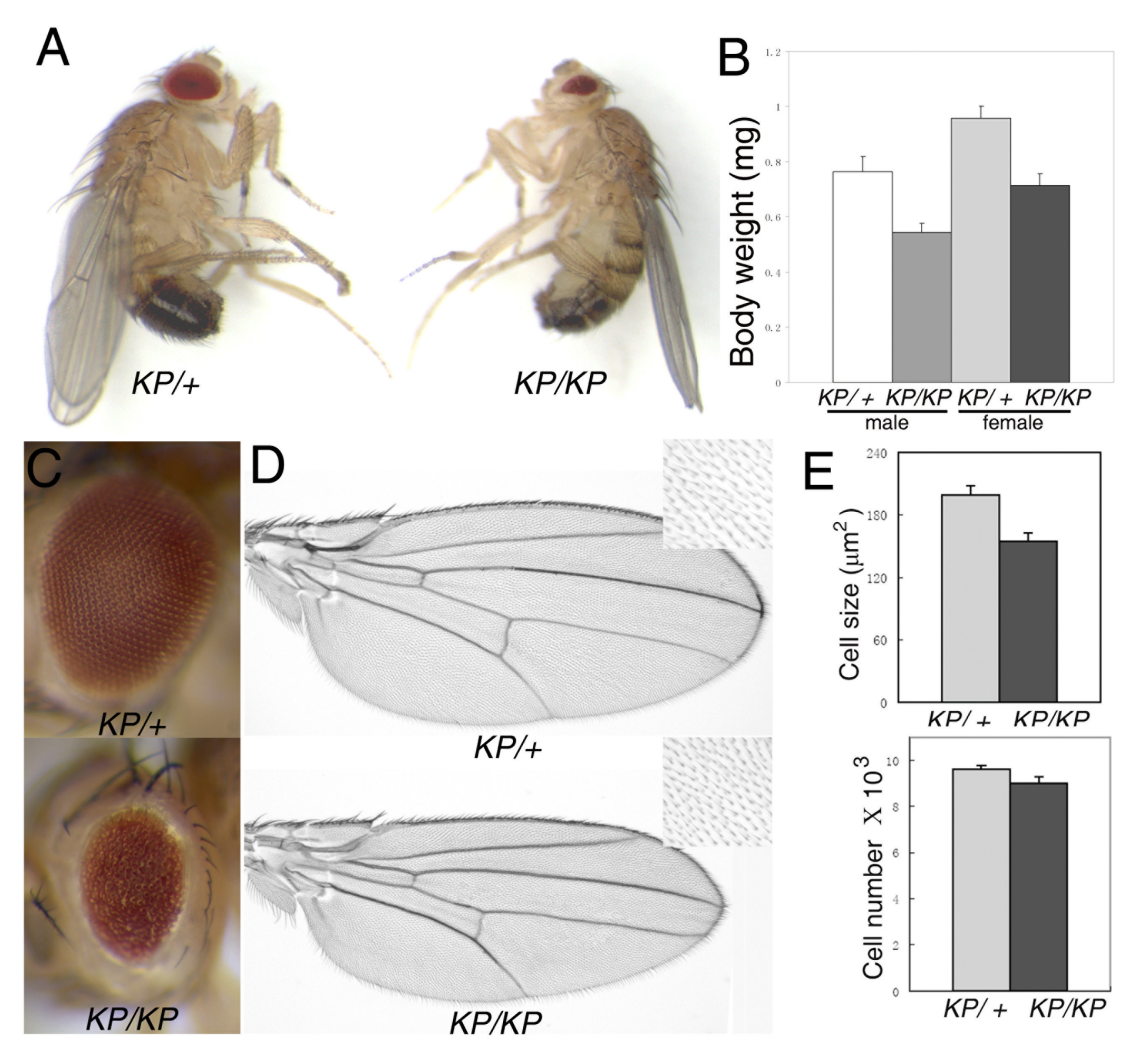
***ras*^*KP *^mutant affects cell size and causes the rough and small eye phenotype**. (A) Comparison of body size of a *ras*^*KP*^/*+ *male (left) and a *ras*^*KP*^/*ras*^*KP *^male (right) 4 days after eclosion. (B) Body weights of *ras*^*KP *^mutants and controls. (C) *ras*^*KP *^homozygous fly eyes are rough and small (bottom panel). (D) Comparison of wing sizes and wing bristle cell density (insets) from *ras*^*KP*^/*ras*^*KP *^(bottom panel) and *ras*^*KP*^/*+ *flies (upper panel). (E) Wing hair size is reduced in *ras*^*KP *^mutant, but the total number of the cells in the wing does not change greatly. (*KP *denotes *ras*^*KP*^). Genotypes: (A) (B) (C) (D) *ras*^*KP*^/*ras*^*KP *^and *ras*^*KP*^/*+*.

We isolated a spontaneous recessive mutation which causes a reduction of body size in the adult flies (Figure 1A). Although about 60% of the mutant homozygotes die at the pupal stage (n>200), there are a small percentage of escapers that survive. Viable mutant females, however, are found to be partial sterile.

**Figure 2:**
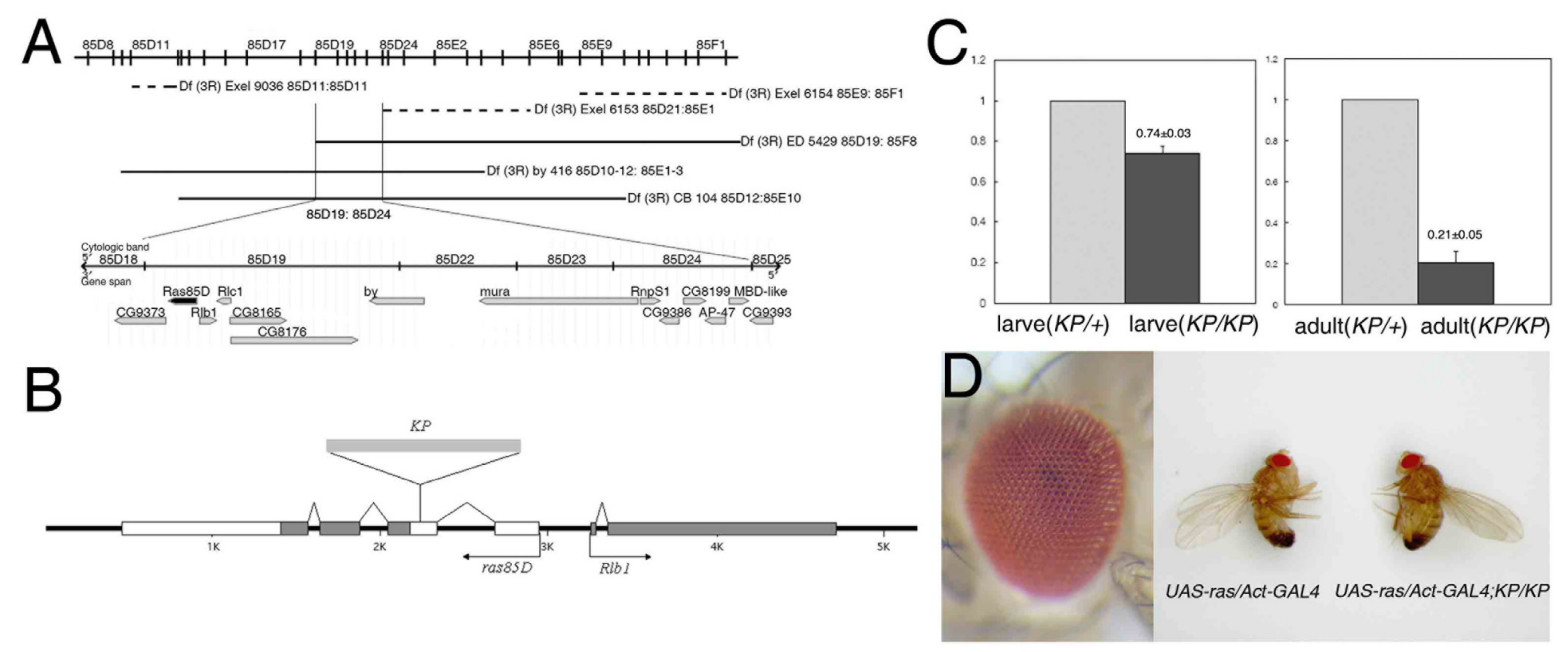
**Identification of a *KP*-element insertion mutation in the *ras *gene**. (A) A cytological map showing the deletions uncovering the mutation to the genomic region spanning the breakpoints at 85D19 and 85D24. Below the map is an overlay of the genomic annotation for this region containing 14 genes . (B) The genomic organization of the *ras *gene showing the position of the KP element insertion. Black boxes indicate coding sequences, white boxes are the UTR sequences, and the arrows indicate the direction of gene transcription. (C) Normalized real-time PCR data where control level of *ras *gene expression is set to 1. *ras *expression level is markedly reduced in the *ras*^*KP*^/*ras*^*KP *^mutant, especially in the adult flies. (D) Constitutive expression of wild-type Ras by the *actin *promoter fully rescues the *ras*^*KP *^mutant eye phenotype (left panel), as well as body size (right panel).

By meiotic recombination and deficiency mapping, we localize the mutation to the cytological position 85D19-24 on the third chromosome, which spans a 50-kb region containing 14 genes  (Figure 2A). PCR amplification and sequencing of exon sequences of these genes from mutant animals reveal a 1165-bp *KP *element insertion in the second exon (5'UTR) of the *Ras1 *gene (or *Ras *oncogene at 85B) (Figure 2B). Quantitative PCR (QPCR) showed *ras *mRNA level was significantly reduced (Figure 2C) in homozygous mutant larvae (~74% of that in heterozygous mutant) and adults (~21% of that in heterozygous mutant). Complementation tests of the mutation with four known *ras *loss-of-function alleles, *ras*^*D*38*N *^[22], *ras85DelB *[23,22], *rasΔC40b*, and *ras ΔC17b *[24], failed to rescue *ras *dependent lethality. These results suggest that the mutation is a partial loss-of-function *ras *allele. This is further supported by the rescue of the mutant phenotype by ubiquitous expression of wild-type *ras *driven by *Act-GAL4 *(Figure 2D). Thus, we named this new mutation as *ras*^*KP*^.

### *ras*^*KP *^mutation reduces wing size mainly by affecting cell size

Although there are no obvious differences in body size between *ras*^*KP *^mutant and wild-type larvae (data not shown), survived mutant adult flies show a significant body weight reduction (Figure 1B and see Additional file 1). Homozygous mutants also have a 23.5% reduction in wing size owing to a decrease in cell size and not cell number (Figure 1D, E).

### Loss of *ras *function causes apoptosis in the *Drosophila *eye

**Figure 3:**
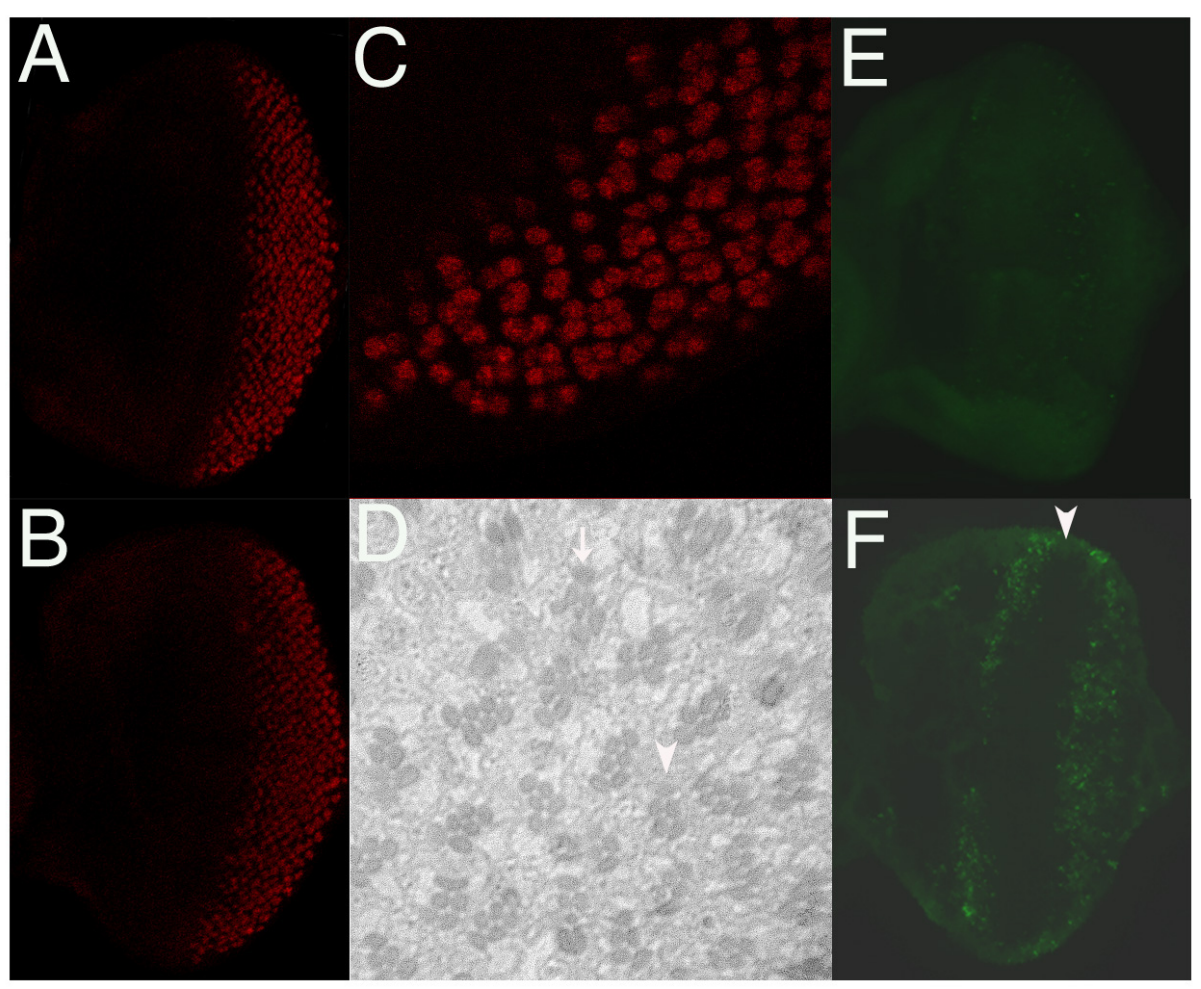
***ras*^*KP *^mutant induces cell death in the eye**. Photoreceptor differentiation, as visualized by anti-Elav staining, occurs normally in third instar larval eye discs of both wild-type (A) and *ras*^*KP *^mutant (B). (C) Enlarge picture of (B) showing details of pattern formation where neuronal cell recruitment and differentiation is not affected in the *ras*^*KP *^mutant. (D) Histological light section of an adult *ras*^*KP *^mutant eye showing a disarrayed pattern of ommatidia. Although the full complement of photoreceptor cell types can be found (arrow), many ommatidia occasionally contain fewer photoreceptor cells (arrow head). Acridine orange staining shows very few apoptotic cells in a wild-type eye disc (E), whereas in the *ras*^*KP *^mutant (F) there is a massive number of dying cells occurring as a band just anterior to the morphogenetic furrow (arrow head) and broadly in the posterior region of the eye disc. Genotypes: (A) (E) wild-type; (B) (C) (D) (F) *ras*^*KP*^/*ras*^*KP*^.

During *Drosophila *eye development, Ras contributes positively to the regulation of cell growth [25] and cell differentiation [26], but negatively to the regulation of apoptosis [9,10]. Such diverse biological effects are thought to be achieved through different levels of Ras activity [27]. *ras*^*KP *^homozygous mutants have small and rough eyes (Figure 1C). To determine the developmental basis of the phenotype, we compare the growth of third instar larval eye imaginal discs of *ras*^*KP *^mutant and wild-type. Both were of similar sizes (data not shown), suggesting that growth was unlikely to be the major reason for the small eye in the mutant adult. Neuronal differentiation in the eye discs, as revealed by anti-Elav antibody staining [28], do occur in *ras*^*KP *^third instar larvae (Figure 3A-C). However, sections of the adult mutant eyes indicate ommatidia are largely disorganized though the majority of them with correct number of out photoreceptor cells and missing the R7 photoreceptor cells (Figure 3D), indicating that differentiation is affected. Acridine orange staining in eye discs of the third instar larvae reveals a substantially increased number of dying cells in mutant animals (Figure 3E, F). Cell death occurs mainly in two regions, a band anterior to the morphogenetic furrow (MF) and a broad region in the posterior part of the eye disc. These results indicate that the rough and small eye phenotype is caused mainly by abnormal apoptosis in the *ras*^*KP *^mutant.

### Apoptosis in *ras*^*KP *^mutant is partially suppressed by disrupting Hid activity

**Figure 4:**
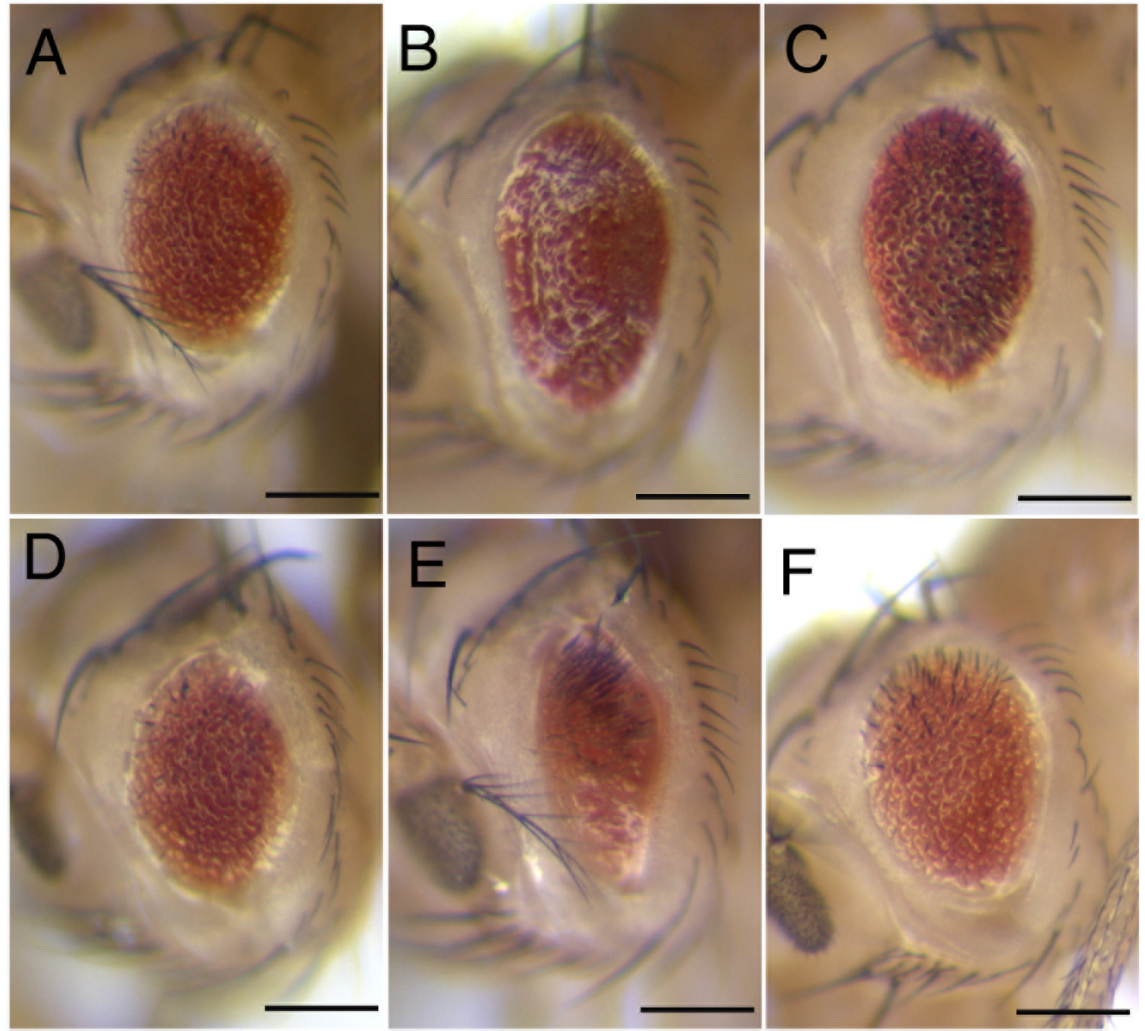
**Genetic interactions of *ras *with genes involved in growth and survival**. (A) *ras*^*KP *^mutant eye is small and rough. (B) *GMR-p35 *only partly suppresses the *ras*^*KP*^-induced cell death. (C) In the *hid *null background, *ras*^*KP*^-induced cell death is also partially suppressed. Ectopic expression of *PI3K *(D), *Akt *(E) and *buffy *(F) have no effect on the *ras*^*KP *^eye phenotype. Genotypes: (A)*GMR-Gal4/CyO; ras*^*KP*^/*ras*^*KP*^, (B)*GMR-p35/CyO; ras*^*KP*^/*ras*^*KP*^, (C)*Df(3L)H99, ras*^*KP*^/*hid*^05014^*ras*^*KP*^, (D)*GMR-Gal4/UAS-p110; ras*^*KP*^/*ras*^*KP*^, (E)*GMR-Gal4/UAS-AKT; ras*^*KP*^/*ras*^*KP*^, (F)*GMR-Gal4/UAS-buffy; ras*^*KP*^/*ras*^*KP*^.

**Figure 5:**
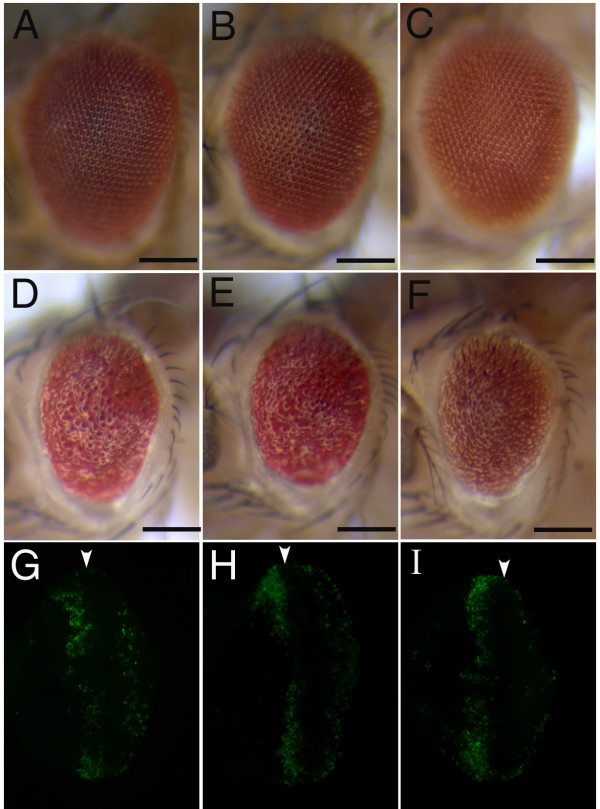
**JNK and Hid cooperate in *ras*^*KP*^-induced cell death**. The reduction of the JNK signaling activity and inhibiting Hid activity by either overexpressing *p35 *or using the *hid *null do not have an effect in the wild-type background (A, B, C). However, the reduction of both activities lead to a strong suppression of the *ras*^*KP *^apoptotic phenotype (D, E, F). When *p35 *or *Hep-IR *is expressed under the GMR promoter in the *ras*^*KP *^mutant eye disc, apoptosis still occurs as detected by acridine orange staining (G and H, respectively). However, when both JNK pathway and Hid activity are inhibited in the *ras*^*KP *^mutant, very few dying cells could be detected (I). Genotypes: (A) *GMR-Gal4/GMR-p35; UAS-DTRAF1-IR*, (B) *GMR-Gal4/GMR-p35; UAS-Hep-IR*, (C) *GMR-Gal4/Cyo; UAS-Hep-IR*, *Df(3L)H99/hid*^05014^, (D) *GMR-Gal4/GMR-p35; UAS-DTRAF1-IR, ras*^*KP*^/*ras*^*KP*^, (E)*GMR-Gal4/GMR-p35; UAS-Hep-IR, ras*^*KP*^/*ras*^*KP*^, (F) *GMR-Gal4/Cyo; UAS-Hep-IR*, *Df(3L)H99, ras*^*KP*^/*hid*^05014^*ras*^*KP *^. (G) *GMR-Gal4/UAS-p35; ras*^*KP*^/*ras*^*KP*^. (H) *GMR-Gal4/Cyo; UAS-Hep-IR, ras*^*KP*^/*ras*^*KP*^. (I) *GMR-Gal4/GMR-p35; UAS-Hep-IR, ras*^*KP*^/*ras*^*KP*^.

In *Drosophila*, Ras has been shown to promote cell survival by both down regulating *hid *expression and inactivating Hid protein through phosphorylation [9,10]. In order to test whether *ras*^*KP*^-induced apoptosis in the eye is mediated by *hid*, we completely removed *hid *function by using a null allele in a *ras*^*KP *^mutant and see if it could suppress the small eye phenotype. We would expect a strong suppression if, indeed, *ras *acts solely on *hid *to promote cell survival. However, as shown in Figure 4C, there is only a marginal suppression as compared to *ras*^*KP *^mutant alone (Figure 4A). Consistent with this result, the expression of the baculovirus caspase inhibitor p35, which has been shown to completely block *hid-*induced apoptosis [11,29], also only partially suppresses the small eye phenotype of *ras*^*KP *^mutant flies (Figure 4B). When assayed for cell death in the eye discs of *GMR-p35; ras*^*KP *^larvae by staining with acridine orange, many apoptotic cells are still observed posterior to the MF (Figure 5G). The data suggests that *ras*^*KP*^-induced cell death is mediated by additional apoptotic pathway independent of *hid *function.

### Inhibiting the JNK pathway appreciably suppresses *ras*^*KP*^-induced apoptosis

To reveal which pathway mediates anti-apoptosis signalling from Ras, we tested for genetic interaction between *ras *and components of several major signalling pathways involved in growth and survival. One prominent candidate is PI3K which has been shown to be a critical effector of Ras in providing an universal survival signal in mammals [30]. PI3K activates Akt which further phosphorylates a number of substrates involved in the regulation of apoptosis [12]. In the fly, there is also evidence to suggest that the activation of the PI3-K/Akt pathway has antiapoptotic activity during embryonic development [9]. To address whether PI3K/Akt activation can rescue the *ras*^*KP*^-induced cell death, we expressed *p110*, which encodes PI3K, or *Akt *in *ras*^*KP *^mutant flies. As shown in Figure 4D and 4E, GMR-driven expression of *PI3K *or *Akt *cannot significantly suppress the *ras*^*KP *^small eye phenotype. Thus, Ras is not likely to transduce the survival signal through PI3-K/Akt in the *Drosophila *eye. We tested another candidate antiapoptotic pathway, which utilizes a *Drosophila *Bcl-2-like protein encoded by the *Buffy *gene [31]. In mammals, the Bcl-2 protein prevents the release of cytochrome c from mitochondria, and consequently inhibits the formation of the Apaf-1 apoptosome [32]. As with *PI3K/Akt*, the overexpression of *Buffy *also cannot suppress the apoptotic eye phenotype of *ras*^*KP *^(Figure 4F).

**Figure 6:**
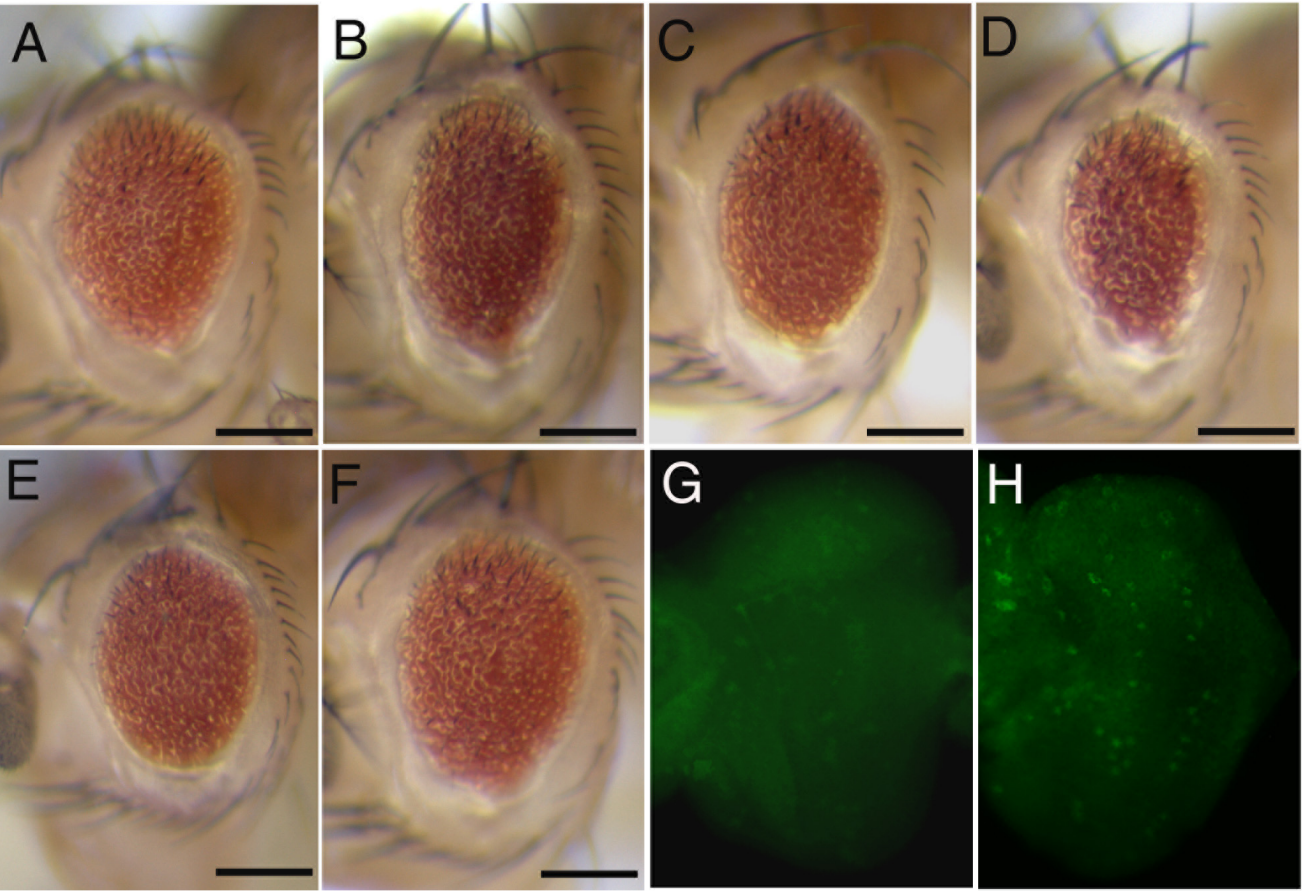
***ras*^*KP*^-induced apoptosis is correlated with JNK signaling**. *ras*^*KP*^-induced cell death could be suppressed by using RNA interference (RNAi) to down regulate the endogenous expression of *DTRAF1 *(A), *dTAK1 *(B), *Hep *(C), *Eiger*(E), and *Wengen *(F). GMR-driven expression of a dominant-negative form of JNK (*Bsk*^*DN*^) also partly suppresses the *ras*^*KP *^eye phenotype (D). Immunostaining eye discs with an anti-phosphorylated JNK antibody reveals an increased level of activated JNK signaling in *ras*^*KP *^mutant (H) as compared to wild-type (G). Genotypes: (A) *GMR-Gal4/UAS-DTRAF1-IR; ras*^*KP*^/*ras*^*KP*^, (B) *GMR-Gal4/CyO; UAS-dTAK-IR, ras*^*KP*^/*ras*^*KP*^, (C)*GMR-Gal4/CyO; UAS-Hep-IR, ras*^*KP*^/*ras*^*KP*^, (D) *GMR-Gal4/CyO; UAS-BskDN, ras*^*KP*^/*ras*^*KP*^, (E) *GMR-Gal4/UAS-Eiger-IR; ras*^*KP*^/*ras*^*KP*^, (F) *GMR-Gal4/UAS-Wengen-IR; ras*^*KP*^/*ras*^*KP*^, (G) wild-type, (H) *ras*^*KP*^/*ras*^*KP*^.

In *Drosophila*, the ERK pathway has been shown to serve as a survival signal that antagonizes JNK signaling from inducing apoptosis [33]. Thus, it follows that, being an upstream activator of ERK, Ras antiapoptotic activity may lie in its ability to inhibit JNK signaling. To address this possibility, we ask whether the *ras*^*KP *^mutant phenotype can be suppressed by the loss of JNK signalling. We take the approach of using RNA interference (RNAi) to down regulate the expression of genes involved in JNK signalling in the *ras*^*KP *^mutant. These genes include *DTRAF1 and DTRAF2 (Drosophila *TRAF proteins), *Hep *(a JNK kinase), and *DTAK1 *(a JNKK Kinase). The reduction of JNK signalling through any of these genes significantly suppresses the apoptotic phenotype of *ras*^*KP *^(Figure 6A-C and data not shown). The genetic interaction is specific for the *ras*^*KP *^allele since the expression of these RNAi constructs by themselves has no phenotypic consequences. In addition to these results, the overexpression a dominant-negative form of *Drosophila *JNK, *Bsk*^*DN*^, also slightly suppresses the apoptosis caused by *ras*^*KP *^(Figure 6D). The activation of the JNK pathway can be achieved by the binding of Eiger to Wengen, which encode, respectively, the mammalian homologs of the tumor necrosis factor TNF and its receptor TNFR [16,17,34,35]. We find that RNAi downregulation of *Eiger *or *Wengen *also partially rescues the apoptotic eye phenotype of the *ras*^*KP *^mutant (Figure 6E, F). As with the regulation of Hid activity by Ras, we ask if JNK activity is also affected. When eye discs from *ras*^*KP *^mutant are stained with an anti-phosphorylated JNK, there is indeed a significant increase in JNK activity (Figure 6H). These data are consistent with the notion that Ras could down regulate JNK activity to inhibit apoptosis.

### JNK and Hid signalling cooperate in *ras*^*KP*^-induced cell death

Since the reduction of either Hid or JNK activity alone can have only a marginal or partial suppression of the *ras*^*KP*^-induced cell death, it raises the possibility that both JNK and Hid activities could cooperate to fully induce the apoptosis associated with *ras*^*KP*^. To address this hypothesis, we simultaneously reduce both Hid and JNK activities in the *ras*^*KP *^eye by co-expression of both p35 to block Hid-dependent cell death and RNAi downregulation of JNK signalling. Indeed, the reduction of both Hid and JNK activity strongly suppresses the *ras*^*KP *^eye phenotype as compared to the suppression by each alone (Figure 5D, E). The suppression was specific to *ras*^*KP*^since the reduction of both activities together in a wild-type background has no phenotypic effect on the eye (Figure 5A, B). The phenotypic eye suppression is further corroborated by the almost complete absence of dying cells in the posterior region of the eye imaginal discs as detected by acridine orange staining (Figure 5I). However, due to the restricted expression pattern of the *GMR *promoter to only postmitotic cells, apoptotic cells anterior to the MF are not affected. It is possible that expression of P35 don't completely block Hid-dependent apoptosis. We thus further show that when JNK signalling is down regulated in the *ras*^*KP*^, *hid*^*null *^double mutant background, the apoptotic eye phenotype is also strongly suppressed (Figure 5F). These data indicate that Ras could inhibit both Hid and JNK-mediated apoptosis.

## Discussion

In this report, we present the genetic evidence that Ras could antagonize JNK signaling, in addition to Hid, to promote cell survival in the developing *Drosophila *eye. Both the genetic interaction and the upregulation of JNK activity in the *ras*^*KP *^mutant support the view that JNK pathway serves as a target of *ras *antiapoptotic activity. *ras*^*KP*^-induced apoptosis in fly eye is significantly suppressed by down regulating JNK pathway components using an RNAi strategy. We further stained the *ras*^*KP *^eye disc with an anti-phospho-JNK antibody and found JNK phosphorylation was increased, which is consistent with the notion that JNK signaling is activated in *ras*^*KP *^eye disc.

Previous work has suggested that the JNK pathway control cell death by regulating *hid *expression [17]. This notion implies that the impact of Ras signaling on the JNK pathway might also converge on Hid activity. In *ras*^*KP*^, *hid*^*null *^double mutant flies with downregulated JNK signaling, the suppression of the apoptotic eye phenotype is much stronger than the suppression when either JNK pathway or Hid activity is inhibited alone. These results indicate that the two pathways have separate effects on cell survival, and that Ras inhibition of JNK-mediated cell death is in part Hid-independent.

Previous work has shown that the forced activation of JNK signaling in the developing eye disc can cause widespread cell death [16,17,36]. However, the normal physiological role of JNK signaling during development is still not clear. The isolation of the *ras*^*KP *^allele has permitted us to examine the role of JNK signaling when it is activated in cells that are deprived of the Ras/MAPK survival signal leading to the induction of apoptosis. Studies using this new loss-of-function *ras *allele will provide further insight into how Ras regulates JNK signaling.

## Conclusion

Ras is known to mediate antiapoptotic signals by inhibiting Hid activity in the *Drosophila *eye. Here we analyze a new *ras *loss-of-function allele, *ras*^*KP*^, which reveals an additional target independent of Hid in the regulation of apoptosis. This new function is likely to be mediated through the JNK pathway since the inhibition of JNK signaling can significantly suppress *ras*^*KP*^-induced apoptosis, whereas the removal of *hid *only weakly suppresses the phenotype. However, the reduction of JNK signaling together with the expression of the baculovirus caspase inhibitor p35, which blocks Hid activity, strongly suppresses the *ras*^*KP *^cell death. In addition, we find a strong correlation between *ras*^*KP*^-induced apoptosis in the eye disc and the activation of JNK signaling. Thus, in the *Drosophila *eye, Ras may protect cells from apoptosis by inhibiting both JNK and Hid activities. Surprisingly, reducing Ras activity in the wing, however, does not cause apoptosis but rather affects cell and organ size. Thus, in addition to its requirement for cell viability, Ras appears to mediate different biological roles depending on the developmental context and on the level of its expression.

## Methods

### Fly Stocks

*Drosophila melanogaster *stocks were raised on standard medium at 25°C. The following stocks were kindly provided by our colleagues. *ras*^*D*38*N *^[22], *rasΔC40b*, *rasΔC17b*, and *pR5.5ΔRlb1 *[24] (Celeste Berg), *UAS-buffy *[31] (Helena Richardson), *Df (3L)H99 *and *Hid*^05014 ^(Kristin White), *UAS-Eiger-IR, UAS-Wengen-IR, UAS-DTRAF1-IR, UAS-DTRAF2-IR, UAS-DTAK1-IR, UAS-Hep-IR*, and *UAS-Bsk*^*DN*^[14] (Tatsushi Igaki). The *UAS-ras*, *ras85DelB*, and all the deficiency lines used in this report were from the Bloomington *Drosophila *Stock Center.

### Mapping and characterization of *ras*^*KP *^allele

The *ras*^*KP *^mutation was mapped to the third chromosome by chromosome segregation. *ras*^*KP *^mutation was further localized by meiotic recombination mapping to between *st *and *cu *using a multiply marked chromosome (*Gap1, th, st, cu, sr, e, and ca*). Deficiency mapping with *Df(3R)ED5429*, *Df(3R)by416*, and *Df(3R)GB104*, narrows the *ras*^*KP *^mutation to the cytological region 85D19-85D24. The open reading frames of a total of 14 genes in this region from the *ras*^*KP *^mutant were PCR amplified and sequenced. The *ras*^*KP *^mutation is found to be an insertion of a 1165 bp fragment of a KP element in the 2^nd ^exon of *ras85D *lying 88 bp upstream of the start codon.

### Analysis of mRNA expression

RNA from adult flies or third instar larvae was prepared using TRIzol Reagent (Invitrogen). RNA was reverse-transcribed with the RNA PCR Kit AMV (TaKaRa). To detect the mRNA level of *ras*, quantitative PCR (QPCR) was carried out using the Brilliant SYBR Green QPCR master mix (STRATAGENE) according to the manufacturer's instruction. A 254 bp target sequence for *ras *was amplified with the forward primer 5'-CCAGAACCATTTCGTGGACG-3' and reverse primer 5'-ACCTCTTCGGCATCCTTTACG-3'. For the internal control, a 247 bp target sequence for *actin *was amplified with the forward primer 5'-GCTCTTCAAAGGCAGCAACCAG-3' and reverse primer 5'-GCACAGCCACGACTCTTACGATTAG-3'.

### Size comparison

All experiments with live flies were conducted at 25°C. For size comparison, embryos were collected every hour to synchronize the developmental stages of flies. Fifty embryos from the same collection were raised in one vial. The animals were reared under identical, uncrowded condition. Relative body weight comparison was done by measuring the weight of a group of flies from either the test genotype (*ras*^*KP*^/*ras*^*KP*^) or its sibling control (*ras*^*KP*^/*+*). Wing areas were measured via Adobe Photoshop 7 (for each genotype, 15 female fly wings were checked). The area of whole wing was measured, exclusive of the alula and the costal cell. Cell density was assessed by counting the number of wing hairs on the dorsal wing surface in a 5000 μm^2 ^area just posterior to the posterior crossvein (PCV).

### Histology and immunofluorescence

Eye disc fixation and staining, as well as adult eye histological section, were performed as described [37]. Acridine orange staining of third instar larval eye discs was performed according to standard procedure [38]. Eye imaginal discs were dissected from third instar larvae in PBS and then fixed in 4% paraformaldehyde in PBST (0.3% Triton X-100 in PBS) for 30 min at room temperature (RT). Blocking was performed by incubating samples in PBST (0.4% Triton X-100 in PBS) with 5% NGS (Normal Goat Serum, *Santa Cruz*) for at least 1 h at RT. The following antibodies were used: rat anti-Elav (1:200) and rabbit anti-active JNK antibody (1:200; *Promega*). Texas Red-conjugated goat anti-rat IgG and FITC-conjugated goat anti-rabbit IgG (1:200; *Santa Cruz*) were used for secondary antibodies, respectively. The stained tissues were analyzed by confocal microscopy (*Leica *TCS-NT).

## Abbreviations

ASK1: Apoptosis signal-regulating kinase 1; DIAP1: *Drosophila *Inhibitor of Apoptosis Protein 1; DTRAF1: *Drosophila melanogaster *tumour-necrosis factor receptor- associated factor 1; EGFR: epidermal growth factor receptor; ERK: extracellular signal-regulated protein kinase; FITC: fluorescein isothiocyanate; GAL4: galactose permease 4; GMR: the eye-specific glass multimer reporter; JNK: c-Jun N-terminal protein kinase; KP: paticular deletion of P element, isolated from the Russian Krasnodar strain; MAPK: mitogen-activated protein kinase; MF: morphogenetic furrow; NGS: Normal Goat Serum; PBST: Phosphate Buffered Saline Tweeen-20; PCV: posterior to the posterior crossvein; PI3K: Phosphoinositide 3-kinase; QPCR: quantitative PCR; RNAi: RNA interference; RT: room template; TNF: tunlor necrosis factor; TNFR: tunlor necrosis factor redceptor; UTR: untranslated region.

## Authors' contributions

YW carried out all of the genetic studies and YW and KJD finished phenotype studies. All authors designed the genetic experiments. YW drafted the manuscript, revised by TX, YZ, HM and KJD. All authors read and approved the final manuscript.

## Supplementary Material

Additional file 1 **The relative eye sizes of all genotypes described in the manuscript**. From females at least eight eyes of each genotype were analyzed. Eye size is measured by NIH Image 1.60. Click here for file

## References

[B1] Adachi-Yamada T, Fujimura-Kamada K, Nishida Y, Matsumoto K (1999). Distortion of proximodistal information causes JNK-dependent apoptosis in *Drosophila *wing. Nature.

[B2] Franke B, Bayatti N, Engele J (2000). Neurotrophins require distinct extracellular signals to promote the survival of CNS neurons *in vitro*. Exp Neurol.

[B3] Raff MC (1992). Social controls on cell survival and cell death. Nature.

[B4] Hay BA, Huh JR, Guo M (2004). The genetics of cell death: approaches, insights and opportunities in *Drosophila*. Nat Rev Genet.

[B5] Brachmann CB, Cagan RL (2003). Patterning the fly eye: the role of apoptosis. Trends Genet.

[B6] Cagan RL, Ready DF (1989). *Notch *is required for successive cell decisions in the developing *Drosophila *retina. Genes Dev.

[B7] Baker NE, Yu SY (2001). The EGF receptor defines domains of cell cycle progression and survival to regulate cell number in the developing *Drosophila *eye. Cell.

[B8] Wang SL, Hawkins CJ, Yoo SJ, Müller HA, Hay BA (1999). The *Drosophila *caspase inhibitor DIAP1 is essential for cell survival and is negatively regulated by HID. Cell.

[B9] Bergmann A, Agapite J, McCall K, Steller H (1998). The *Drosophila *gene *hid *is a direct molecular target of Ras-dependent survival signaling. Cell.

[B10] Kurada P, White K (1998). Ras promotes cell survival in *Drosophila *by downregulating *hid *expression. Cell.

[B11] Grether ME, Abrams JM, Agapite J, White K, Steller H (1995). The head involution defective gene of *Drosophila melanogaster *functions in programmed cell death. Genes Dev.

[B12] Datta SR, Brunet A, Greenberg ME (1999). Cellular survival: a play in three Akts. Genes Dev.

[B13] Davis RJ (2000). Signal transduction by the JNK group of MAP kinases. Cell.

[B14] Igaki T, Pagliarini RA, Xu T (2006). Loss of cell polarity drives tumor growth and invasion through JNK activation in *Drosophila*. Curr Biol.

[B15] Stronach B (2005). Dissecting JNK signaling, one KKKinase at a time. Dev Dyn.

[B16] Igaki T, Kanda H, Yamamoto-Goto Y, Kanuka H, Kuranaga E, Aigaki T, Miura M (2002). *Eiger*, a TNF superfamily ligand that triggers the *Drosophila *JNK pathway. Embo J.

[B17] Moreno E, Yan M, Basler K (2002). Evolution of TNF signaling mechanisms: JNK-dependent apoptosis triggered by Eiger, the *Drosophila *homolog of the TNF superfamily. Curr Biol.

[B18] Goberdhan DC, Wilson C (1998). JNK, cytoskeletal regulator and stress response kinase? A *Drosophila *perspective. Bioessays.

[B19] Xia Z, Dickens M, Raingeaud J, Davis RJ, Greenberg ME (1995). Opposing effects of ERK and JNK-p38 MAP kinases on apoptosis. Science.

[B20] Chen J, Fujii K, Zhang L, Roberts T, Fu H (2001). Raf-1 promotes cell survival by antagonizing apoptosis signal-regulating kinase 1 through a MEK-ERK independent mechanism. Proc Natl Acad Sci USA.

[B21] Bradley JR, Pober JS (2001). Tumor necrosis factor receptor-associated factors (TRAFs). Oncogene.

[B22] Simon MA, Bowtell DD, Dodson GS, Laverty TR, Rubin GM (1991). Ras1 and a putative guanine nucleotide exchange factor perform crucial steps in signaling by the sevenless protein tyrosine kinase. Cell.

[B23] Doyle HJ, Bishop JM (1993). Torso, a receptor tyrosine kinase required for embryonic pattern formation, shares substrates with the sevenless and EGF-R pathways in *Drosophila*. Genes Dev.

[B24] Schnorr JD, Berg CA (1996). Differential activity of Ras1 during patterning of the *Drosophila *dorsoventral axis. Genetics.

[B25] Karim FD, Rubin GM (1998). Ectopic expression of activated Ras1 induces hyperplastic growth and increased cell death in *Drosophila *imaginal tissues. Development.

[B26] Fortini ME, Simon MA, Rubin GM (1992). Signaling by the *sevenless *protein tyrosine kinase is mimicked by Ras1 activation. Nature.

[B27] Halfar K, Rommel C, Stocker H, Hafen H (2001). Ras controls growth, survival and differentiation in the *Drosophila *eye by different thresholds of MAP kinase activity. Development.

[B28] Robinow S, White K (1991). Characterization and spatial distribution of the ELAV protein during *Drosophila *melanogaster development. J Neurobiol.

[B29] Hay BA, Wolff T, Rubin GM (1994). Expression of baculovirus P35 prevents cell death in *Drosophila*. Development.

[B30] Downward J (1998). Ras signaling and apoptosis. Curr Opin Genet Dev.

[B31] Quinn L, Coombe M, Mills K, Daish T, Colussi P, Kumar S, Richardson H (2003). Buffy, a *Drosophila *Bcl-2 protein, has anti-apoptotic and cell cycle inhibitory functions. Embo J.

[B32] Yang J, Liu X, Bhalla K, Kim CN, Ibrado AM, Cai J, Peng TI, Jones DP, Wang X (1997). Prevention of apoptosis by Bcl-2: release of cytochrome c from mitochondria blocked. Science.

[B33] Adachi-Yamada T, Gotoh T, Sugimura I, Tateno M, Nishida Y, Onuki T, Date H (1999). *De novo *synthesis of sphingolipids is required for cell survival by down-regulating c-Jun N-terminal kinase in *Drosophila *imaginal discs. Mol Cell Biol.

[B34] Kanda H, Igaki T, Kanuka H, Yagi T, Miura M (2002). Wengen, a member of the *Drosophila *tumor necrosis factor receptor superfamily, is required for Eiger signaling. J Biol Chem.

[B35] Kauppila S, Maaty WSA, Chen P, Tomar RS, Eby MT, Chapo J, Chew S, Rathore N, Zachariah S, Sinha SK, Abrams JM, Chaudhary PM (2003). Eiger and its receptor, Wengen, comprise a TNF-like system in *Drosophila*. Oncogene.

[B36] Takatsu Y, Nakamura M, Stapleton M, Danos MC, Matsumoto K, O'Connor MB, Shibuya H, Ueno N (2000). TAK1 participates in c-Jun N-terminal kinase signaling during *Drosophila *development. Mol Cell Biol.

[B37] Xu T, Harrison SD (1994). Mosaic analysis using FLP recombinase. Methods Cell Biol.

[B38] Spreij TE (1970). Cell death during the development of the imaginal discs of *Calliphora erythrocephala*. Netherlands J Zool.

