# Supplementary material for: Ras promotes cell survival by antagonizing both JNK and Hid signals in the Drosophila eye
Source: BMC Dev Biol. 2009 Oct 20;9:53. doi: 10.1186/1471-213X-9-53 (PMC2773777; doi:10.1186/1471-213X-9-53)
Supplement: Additional file 1 — The relative eye sizes of all genotypes described in the manuscript. From females at least eight eyes of each genotype were analyzed. Eye size is measured by NIH Image 1.60. [file 1471-213X-9-53-S1.DOC]

**Additional file 1:** The relative eye sizes of all genotypes described in the manuscript.

| **Genotype** | **Eye size** |
| --- | --- |
| *GMR-Gal4* | 1.000±0.069 |
| *GMR-Gal4/CyO; ras^KP^/ras^KP^* | 0.324±0.037 |
| *GMR-p35/CyO; ras^KP^/ras^KP^* | 0.408±0.016 |
| *Df(3L)H99, ras^KP^/hid^05014^ ras^KP^* | 0.451±0.014 |
| *GMR-Gal4/USA-p110; ras^KP^/ras^KP^* | 0.318±0.008 |
| *GMR-Gal4/UAS-AKT; ras^KP^/ras^KP^* | 0.254±0.007 |
| *GMR-Gal4/UAS-buffy; ras^KP^/ras^KP^* | 0.384±0.012 |
| *GMR-Gal4/UAS-DTRAF1-IR; ras^KP^/ras^KP^* | 0.479±0.028 |
| *GMR-Gal4/CyO; UAS-dTAK-IR, ras^KP^/ras^KP^* | 0.436±0.020 |
| *GMR-Gal4/CyO; UAS-Hep-IR, ras^KP^/ras^KP^* | 0.463±0.018 |
| *GMR-Gal4/CyO; UAS-BskDN, ras^KP^/ras^KP^* | 0.341±0.007 |
| *GMR-Gal4/UAS-Eiger-IR; ras^KP^/ras^KP^* | 0.413±0.013 |
| *GMR-Gal4/UAS-Wengen-IR; ras^KP^/ras^KP^* | 0.492±0.029 |
| *GMR-Gal4/GMR-p35; UAS-DTRAF1-IR* | 1.010±0.032 |
| *GMR-Gal4/GMR-p35; UAS-Hep-IR* | 0.992±0.007 |
| *GMR-Gal4/CyO; UAS-Hep-IR, Df(3L)H99/hid^05014^* | 0.994±0.014 |
| *GMR-Gal4/GMR-p35; UAD-DTRAF1-IR, ras^KP^/ras^KP^* | 0.556±0.040 |
| *GMR-Gal4/GMR-p35; UAS-Hep-IR, ras^KP^/ras^KP^* | 0.574±0.044 |
| *GMR-Gal4/CyO; UAS-Hep-IR, Df(3L)H99, ras^KP^/hid^05014^, ras^KP^* | 0.566±0.054 |

From females at least eight eyes of each genotype were analyzed. Eye size is measured by NIH Image 1.60.
